# Weather and children’s physical activity; how and why do relationships vary between countries?

**DOI:** 10.1186/s12966-017-0526-7

**Published:** 2017-05-30

**Authors:** Flo Harrison, Anna Goodman, Esther M. F. van Sluijs, Lars Bo Andersen, Greet Cardon, Rachel Davey, Kathleen F Janz, Susi Kriemler, Lynn Molloy, Angie S Page, Russ Pate, Jardena J Puder, Luis B Sardinha, Anna Timperio, Niels Wedderkopp, Andy P. Jones, on behalf the ICAD collaborators, LB Andersen, S Anderssen, G Cardon, A Cooper, R Davey, U Ekelund, DW Esliger, K Froberg, P Hallal, KF Janz, K Kordas, S Kriemler, A Page, R Pate, JJ Puder, J Reilly, J Salmon, LB Sardinha, LB Sherar, A Timperio, EMF van Sluijs

**Affiliations:** 1 Norwich Medical School, University of East Anglia, Norwich, UK; 2 Faculty of Epidemiology and Public Health, London School of Hygiene and Tropical Medicine, Keppel Street, London, UK; 3 MRC Epidemiology Unit, University of Cambridge School of Clinical Medicine, Box 285 Institute of Metabolic Science, Cambridge Biomedical Campus, Cambridge, CB2 0QQ UK; 4 UKCRC Centre for Diet and Activity Research, University of Cambridge School of Clinical Medicine, Institute of Metabolic Science, Cambridge, UK; 5 Norwegian School of Sport Science, Oslo, Norway, and Faculty of Teacher Education and Sport, Sogn og Fjordane University College, Førde, Norway; 6 Department of Movement and Sports Sciences, Ghent University, 9000 Ghent, Belgium; 7 Centre for Research & Action in Public Health, Health Research Institute, University of Canberra, Canberra, Australia; 8 Department of Health and Human Physiology, University of Iowa, Iowa City, USA; 9 Epidemiology, Biostatistics and Public Health Institute, University of Zürich, Zürich, Switzerland; 10 School of Social and Community Medicine, University of Bristol, Bristol, UK; 11 Centre for Exercise, Nutrition and Health Sciences, University of Bristol, Bristol, UK; 12 Department of Exercise Science, University of South Carolina, Columbia, USA; 13 Service of Endocrinology, Diabetes and Metabolism & Division of Pediatric Endocrinology, Diabetes and Obesity, Centre Hospitalier Universitaire Vaudois, University of Lausanne, Lausanne, Switzerland; 14 Exercise and Health Laboratory, Department of Sports and Health, CIPER, Faculdade de Motricidade Humana, Universidade de Lisboa, Cruz-Quebrada, Lisbon, Portugal; 15 Centre for Physical Activity and Nutrition Research, Deakin University, Melbourne, Australia; 16 University of Southern Denmark, Odense, Denmark

**Keywords:** ICAD, Child, Adolescent, Physical activity, Season, Weather

## Abstract

**Background:**

Globally most children do not engage in enough physical activity. Day length and weather conditions have been identified as determinants of physical activity, although how they may be overcome as barriers is not clear. We aim to examine if and how relationships between children’s physical activity and weather and day length vary between countries and identify settings in which children were better able to maintain activity levels given the weather conditions they experienced.

**Methods:**

In this repeated measures study, we used data from 23,451 participants in the International Children’s Accelerometry Database (ICAD). Daily accelerometer-measured physical activity (counts per minute; cpm) was matched to local weather conditions and the relationships assessed using multilevel regression models. Multilevel models accounted for clustering of days within occasions within children within study-cities, and allowed us to explore if and how the relationships between weather variables and physical activity differ by setting.

**Results:**

Increased precipitation and wind speed were associated with decreased cpm while better visibility and more hours of daylight were associated with increased cpm. Models indicated that increases in these variables resulted in average changes in mean cpm of 7.6/h of day length, −13.2/cm precipitation, 10.3/10 km visibility and −10.3/10kph wind speed (all *p* < 0.01). Temperature showed a cubic relationship with cpm, although between 0 and 20 degrees C the relationship was broadly linear. Age showed interactions with temperature and precipitation, with the associations larger among younger children. In terms of geographic trends, participants from Northern European countries and Melbourne, Australia were the most active, and also better maintained their activity levels given the weather conditions they experienced compared to those in the US and Western Europe.

**Conclusions:**

We found variation in the relationship between weather conditions and physical activity between ICAD studies and settings. Children in Northern Europe and Melbourne, Australia were not only more active on average, but also more active given the weather conditions they experienced. Future work should consider strategies to mitigate the impacts of weather conditions, especially among young children, and interventions involving changes to the physical environment should consider how they will operate in different weather conditions.

## Background

Physical inactivity increases the risk of many non-communicable diseases and has been recognised as a major contributor to the global burden of ill health [1]. It is therefore worrying that in many high-income settings average time spent in moderate to vigorous physical activity (MVPA) is well below the recommendation of 60 min per day [2]. A recent study in the UK, for example found that only 51% of 7 year olds met this target [3]. Understanding the barriers and drivers of physical activity in children is key to developing sustainable and successful interventions to increase activity levels. It has been consistently observed that children’s activity levels exhibit a seasonal pattern. This has been reported in many settings including Europe [4–12], the USA [13–15] and Australia [16]. Activity levels are generally lowest in the winter, when dark evenings and cool, wet weather is thought to inhibit activity [17]. Understanding how day length and weather influence physical activity is therefore a useful step in the development of sustainable interventions to maintain activity levels throughout the year.

Several studies have found relationships between different weather variables and children’s physical activity. Rainfall has been associated with decreased activity [18–22]. For example, Harrison and colleagues analysed a sample of 1794 9–10 year old English children and found they undertook almost 15 min less MVPA on the wettest days compared to days with no rain [20]. Conversely, temperature has shown a positive association with physical activity, with small to moderate increases in step counts associated with a 10 °C increase in temperature in studies in New Zealand (*n* = 1115) [21] and Canada (*n* = 1293) [18]. In addition to these relatively simple associations, there are suggestions that certain combinations of weather variables may have different associations with physical activity in adults [23], and that the relationships seen between weather conditions and physical activity may vary with age [20]. However, studies examining weather and physical activity have tended to be confined to small areas, often a single city or state, which limits variability in exposure measures. Although the weather varies daily within a single location, heterogeneity of weather exposures is often low in single site studies within a limited time frame. Further, the different means by which both physical activity and weather can be measured makes it difficult to compare their relationships with physical activity across different settings and populations. This is problematic because understanding if and how children in different settings respond to similar weather conditions might help us to identify potential adaptive strategies. For example, there may be settings where the physical environment supports outdoor active play in wet weather, or where the cultural environment promotes activity even in the cold. The fact that seasonal patterns in physical activity [24] and association with day length [25] are not consistently observed in all settings suggests that there may be some cultural adaptations to the markers of seasons; weather and day length.

The pooling of objectively measured physical activity from studies conducted around the world in the International Children’s Accelerometry Database (ICAD) provides the opportunity to explore the relationships between weather and physical activity over a wide range of exposures, and understand if and how these relationships differ by setting (e.g. the city/country in which studies are located) and participants.

Using the ICAD data, this paper aims to answer the following questions:

1. What is the relationship between day length, weather conditions and physical activity, including potential interactions between weather variables?
2. How do the relationships between day length, weather conditions and physical activity vary between settings?
3. In which settings do children appear to best maintain their physical activity levels given the weather conditions they are exposed to?

## Methods

### Study design and participants

**Fig. 1:**
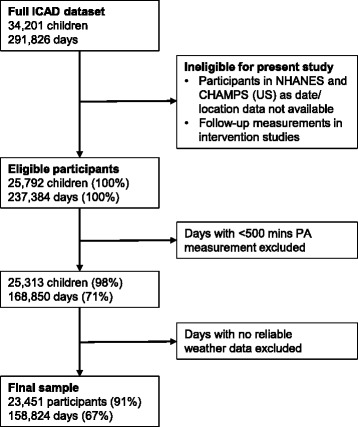
Flow chart of the study sample selection

ICAD pooled physical activity and demographic data from studies worldwide. ICAD’s methods are explained in full elsewhere [26], and so are only briefly described here. Between 2008 and 2010, raw accelerometery files were obtained from 21 studies that had measured physical activity with Actigraph accelerometers. The ICAD studies included cross-sectional, longitudinal, and intervention designs. Participants ranged in age from 3 to 18 years, and the dates of the studies ranged from 1997 to 2009. Details of the study characteristics are given in Table 1. Physical activity and demographic data were standardised and reduced using consistent methods, providing a sample of 34,201 individual participants from 10 countries. However, not all participants in ICAD were eligible for this study. Specifically those taking part in a study that did not collect date and location data and follow-up samples from intervention studies were not eligible, leaving an eligible sample of 25,792 individuals (see Fig. 1). Participant age, sex, height, and weight were available from all participating studies. Body mass index was derived from height and weight measurements and was used to classify participants as normal weight or overweight/obese based on international age and sex specific cut points [27].

**Table 1:** Characteristics of study participants

						Participant Characteristics. N (% of study)					
						Gender^c^	Age band			Weight status^d^	
Study	Location	Months	Study Type^a^	N Participants^b^	N days^b^	Female	3–5 years	6–12 years	13–18 years	Over weight	Obese
ALSPAC	Bristol, UK	Jan-Dec*	Long	6613 (28%)	61,101 (38%)	3449 (52%)	0 (0%)	4202 (64%)	2411 (36%)	1094 (17%)	314 (5%)
Ballabeina	Aarau, Switzerland	Aug-Sep	Int	375 (2%)	1496 (1%)	185 (49%)	336 (90%)	39 (10%)	0 (0%)	29 (8%)	14 (4%)
Belgium Pre-School Study	Antwerp, Belgium	Feb-Mar, Oct-Dec	CS	430 (2%)	1303 (1%)	225 (52%)	411 (96%)	19 (4%)	0 (0%)	50 (12%)	56 (13%)
CHAMPS (UK)	Stoke-on-Trent, UK	Nov-Mar, May	CS	529 (2%)	2399 (2%)	257 (49%)	82 (16%)	291 (55%)	156 (29%)	96 (18%)	38 (7%)
CLAN	Melbourne, Australia	Jul-Dec*	Long	1115 (5%)	9426 (6%)	593 (53%)	54 (5%)	812 (73%)	249 (22%)	241 (22%)	100 (9%)
CoSCIS	Copenhagen, Denmark	Dec-Jun*	Int	674 (3%)	3523 (2%)	317 (47%)	4 (1%)	670 (99%)	0 (0%)	78 (12%)	29 (4%)
EYHS Denmark	Copenhagen, Denmark	Jan-Dec*	Long	1365 (6%)	7223 (5%)	755 (55%)	0 (0%)	790 (58%)	575 (42%)	172 (13%)	28 (2%)
EYHS Estonia	Tartu, Estonia	Aug-May*	CS	651 (3%)	2246 (1%)	360 (55%)	0 (0%)	335 (51%)	316 (49%)	55 (8%)	6 (1%)
EYHS Norway	Oslo, Norway	Feb, Apr-Jun, Oct, Nov	CS	248 (1%)	650 (0%)	120 (48%)	0 (0%)	248 (100%)	0 (0%)	22 (9%)	2 (1%)
EYHS Portugal	Funchal, Madeira	Jan-Jul*	Long	748 (3%)	1950 (1%)	388 (52%)	0 (0%)	462 (62%)	286 (38%)	130 (17%)	52 (7%)
HEAPS	Melbourne, Australia	Feb-Dec*	Long	1368 (6%)	8219 (5%)	723 (53%)	276 (20%)	1003 (73%)	89 (7%)	283 (21%)	120 (9%)
Iowa Bone Development Study	Des Moines, USA	Sep-Nov	Long	604 (3%)	7300 (5%)	305 (50%)	75 (12%)	429 (71%)	100 (17%)	95 (16%)	139 (23%)
KISS	Aarau, Switzerland	Aug-Sep, Nov	Int	427 (2%)	2334 (1%)	219 (51%)	0 (0%)	427 (100%)	0 (0%)	48 (11%)	33 (8%)
MAGIC	Glasgow, UK	Sep-Nov	CS	429 (2%)	2080 (1%)	215 (50%)	429 (100%)	0 (0%)	0 (0%)	70 (16%)	17 (4%)
PEACH	Bristol, UK	Jan-Apr, Jun, Jul, Sep-Dec *	Long	1204 (5%)	10,011 (6%)	619 (51%)	0 (0%)	1204 (100%)	0 (0%)	216 (18%)	71 (6%)
SPEEDY	Norwich, UK	Feb-Jul	CS	1977 (8%)	9761 (6%)	1101 (56%)	0 (0%)	1977 (100%)	0 (0%)	358 (18%)	116 (6%)
Project TAAG	Columbia (SC), USA	Jan-Mar*	Int	754 (3%)	4070 (3%)	754 (100%)	0 (0%)	203 (27%)	551 (73%)	164 (22%)	127 (17%)
	Minneapolis, USA	Jan-Apr*		880 (4%)	5633 (4%)	880 (100%)	0 (0%)	177 (20%)	703 (80%)	159 (18%)	69 (8%)
	New Orleans, USA	Jan-Apr*		693 (3%)	3907 (2%)	693 (100%)	0 (0%)	200 (29%)	493 (71%)	171 (25%)	136 (20%)
	San Diego, USA	Jan-Apr*		869 (4%)	5185 (3%)	869 (100%)	0 (0%)	212 (24%)	657 (76%)	193 (22%)	138 (16%)
	Tucson, USA	Jan-Apr*		653 (3%)	4113 (3%)	653 (100%)	0 (0%)	234 (36%)	419 (64%)	127 (19%)	59 (9%)
	Washington DC, USA	Jan-May*		845 (4%)	4894 (3%)	845 (100%)	0 (0%)	194 (23%)	651 (77%)	169 (20%)	111 (13%)
All				23,451 (100%)	158,824 (100%)	14,525 (62%)	1667 (7%)	14,128 (60%)	7656 (33%)	4020 (17%)	1775 (8%)

* Study collected valid physical activity data for at least 1/3 of the year (120 days)

^a^Abbreviations for study type: *Long* Longitudinal, *Int* Intervention, *CS* Cross-sectional

^b^Number in individual study, and % contribution to total sample

^c^ref. Male

^d^ref. not overweight or obese (including underweight)

### Physical activity measurements

All studies within ICAD used waist-worn uniaxial accelerometers made by Actigraph; either the 7164 or the GTM1 models. Accelerometer sampling frequency ranged from 5 to 60 s, and so all were re-integrated to 60 s. Non-wear time was defined as a period of 60 min of consecutive zeros allowing for 2 minutes of non-zeros, and a valid measurement day was defined as one on which at least 500 min of wear time were recorded, in line with previous work [19, 20, 25]. Physical activity measurements were taken over up to seven consecutive days at each measurement occasion. For the longitudinal and intervention studies, there were up to four measurement occasions per child. The outcome measure for these analyses was daily mean daytime (7 am-9 pm) counts per minute (cpm), a measure of overall physical activity. We chose cpm as an outcome rather than time spent in MVPA in order to capture physical activity across a full range of intensities. We did perform some sensitivity testing, finding similar results when repeating our main analysis with time spend in MVPA (≥3000 cpm [28]) as an outcome.

### Exposure measures

Weather data were obtained from the National Oceanic and Atmospheric Administration’s Global Summary of the Day (GSOD) [29] station data. GSOD provides daily summaries of temperature (°C), precipitation (mm), wind speed (kph), visibility (km) and snow depth (mm) along with individual stations’ reporting practices. The information on reporting practices indicated where a zero value could be separated from missing data. Temperature, precipitation, wind speed and snow depth have all been associated with children’s physical activity in past work [18–22, 30]. Visibility is a measure of how far a human can see given that day’s conditions. It is estimated automatically by a sensor which determines the distance a beam of light can travel before its luminous flux is reduced to prescribed value (typically 5% of its original value). The visibility on a given day is determined by a number of climatic factors including sunshine, cloud cover and haze [31], which have also been associated with physical activity [21, 32]. Each day of physical activity measurement was linked to the nearest GSOD weather station reporting weather data for that day. If there was no weather station within 50 km of the study location reporting on a given day, then that day’s physical activity measurements in the study were excluded from the analysis. The GSOD stations used ranged from 1.3 km to 37 km from the study locations (mean 9 km). Day length was defined as the time between sunrise and sunset. This is mathematically derived based on the date and geographic location. We obtained day lengths for each measurement day/location from https://www.timeanddate.com/sun//, based on physical activity measurement dates and the location of each study.

### Inclusion/exclusion criteria

A number of ICAD participants were excluded from these analyses due to the non-availability of physical activity data and weather data. Figure 1 details the numbers excluded and reasons for their exclusion. The analyses presented in this paper were conducted at the day level rather than being aggregated to participant means, because weather varies from 1 day to the next. Measurement days with ≥500 mins wear time (a threshold in line with previous work [20, 25, 33]) were excluded (*n* = 68,534 days). Measurement days were dropped where they could not be matched to reliable weather data. Reliability of weather data was deemed insufficient where the differences between zero and ‘no data’ could not be determined based on the reporting information recorded in GSOD. Unfortunately, this included all measurements from the Pelotas study. The final sample included in these analyses therefore comprised 158,824 measurement days from 23,451 participants in 17 studies. Within this sample measurements were taken throughout the year.

### Statistical analysis

A multilevel modelling approach was used to explore the associations between weather conditions and cpm. The structure of ICAD is strongly hierarchical with days clustered within occasions within children. The participants themselves were then nested within both studies and cities. Three cities each hosted two studies, and one study was located across six different cities. The top level in the hierarchy was therefore modelled as study-city pairs (‘settings’, *N* = 22). In all models, the outcome (cpm) was log transformed to improve normality.

As both the outcome (cpm) and exposure measures (weather and day length) were temporally auto-correlated, multilevel mixed-effects linear regression was specified with an auto-correlated residual structure based on date. In addition, one-day lag terms for the weather variables were included in all models. The previous day’s weather may impact physical activity on the current day either via compensation (e.g. children unable to play outdoors because of wet weather 1 day being more active the next), or via the impact on environmental conditions (e.g. wet surfaces inhibiting outdoor play).

To examine the relationships between the weather variables and cpm, we built a model including all of the weather variables and day length along with the demographic and measurement covariates: wear time, age, sex, weight status, and weekend vs weekday. The shape of the relationships between each weather variable and physical activity was explored by plotting the residuals from a model containing only the covariates against each of the weather variables and day length, and where indicated non-linear terms were fitted. Models were run with paired interactions between each combination of the following six variables: age, day length, temperature, precipitation, visibility and wind speed. Where interactions were statistically significant (*p* < 0.01), stratified models were run. Age was included as a potential effect modifier given the differences we have previously observed in the impact of rainfall on physical activity as children age [20].

To explore if and how the relationships between weather variables and physical activity differ by setting, the weather variables were added one at a time to the random part of the model at the setting level. Estimates of the random effects were used to plot the slopes and intercepts of the relationships by setting. To explore differences in the effect of weather on physical activity by setting, the random effects (intercepts) at the setting level of the main model were examined to see which settings appeared to have more active children compared to what would be expected based on the participant characteristics, weather conditions and day length.

## Results

**Fig. 2:**
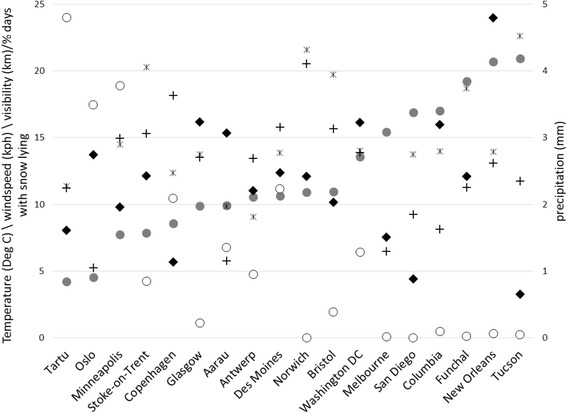
Summary of weather conditions over the data collection period by city. Variables shown are mean daily Temperature (°C; ), mean daily wind speed (kph; **+**), mean daily visibility (km; ), % of measurement days with snow lying on the ground (○), and mean daily precipitation (mm; ◆)

Table 1 shows the characteristics of the participants included in these analyses. Overall there were more female than male participants in these analyses (as well as in ICAD generally), due largely to the inclusion of the all-female Project TAAG. Figure 2 shows mean weather conditions on measurement days at the included cities. It shows that the cities included experience a wide range of weather conditions, including cold and snowy (Tartu/Oslo), hot and dry (Tucson), hot and wet (New Orleans), and a group of cities with a mean temperature around 11 °C and mean precipitation of 2–2.5 mm (Antwerp / Des Moines / Norwich / Bristol).

### QU1) what is the relationship between physical activity and day length/weather, including potential interactions?

Table 2 shows the results of the models of log cpm between 7 am and 9 pm. The adjusted univariate models show statistically significant associations between all of the weather variables/day length and log cpm. Increased precipitation and wind speed were associated with decreased cpm while better visibility and longer days were associated with increased cpm. Examination of the residuals from the models with covariates only indicated mostly linear trends, but a more complex, cubic relationship between cpm and temperature. Most of the weather variables showed low to moderate correlation between each other with correlation coefficients ranging from −0.17 to 0.24. Day length and temperature were more strongly correlated (*r* = 0.56). One-day lag terms for most of the weather variables were also statistically significant in these models, and showed improved fit. For temperature and precipitation measurements on the previous day showed associations in the same direction as values for the current day. For visibility, current day and one-day lag values showed associations in different directions.

**Table 2:** Results of multilevel model of log cpm 7 am-9 pm

	Univariate associations^a^				Fully adjusted model			
	β	lower	upper	*p*	β	lower	upper	*p*
Wear time 7 am-9 pm (hours)					0.021	0.020	0.023	**<0.001**
Age (years)					−0.054	−0.056	−0.053	**<0.001**
Sex (Female)					−0.174	−0.183	−0.165	**<0.001**
Overweight/obese (vs normal weight)					−0.051	−0.060	−0.042	**<0.001**
Weekend (vs weekday)					−0.038	−0.043	−0.034	**<0.001**
Day length (hours)	0.021	0.019	0.022	**<0.001**	0.015	0.014	0.017	**<0.001**
Temperature (10 deg. C)	0.060	0.046	0.073	**<0.001**	0.057	0.043	0.071	**<0.001**
Temperature ^2^	0.020	0.012	0.028	**<0.001**	0.013	0.004	0.021	**0.004**
Temperature ^3^	−0.009	−0.013	−0.006	**<0.001**	−0.009	−0.013	−0.006	**<0.001**
One day lag Temperature	0.013	0.000	0.027	*0.057*	−0.012	−0.026	0.002	*0.100*
One day lag Temperature ^2^	0.013	0.005	0.022	**0.002**	0.010	0.001	0.018	0.031
One day lag Temperature ^3^	−0.010	−0.013	−0.007	**<0.001**	−0.006	−0.009	−0.002	**0.002**
Precipitation (cm)	−0.034	−0.038	−0.031	**<0.001**	−0.027	−0.031	−0.023	**<0.001**
One day lag Precipitation	−0.007	−0.010	−0.003	**0.001**	−0.007	−0.011	−0.003	**0.001**
Visibility (10 km)	0.024	0.021	0.027	**<0.001**	0.021	0.018	0.024	**<0.001**
One day lag Visibility	−0.004	−0.007	−0.001	**0.006**	−0.008	−0.011	−0.005	**<0.001**
Wind speed (10 kph)	−0.022	−0.026	−0.019	**<0.001**	−0.021	−0.025	−0.018	**<0.001**
One day lag Wind speed	0.002	−0.001	0.005	*0.264*	0.003	0.000	0.007	*0.084*
Snow lying on ground? (vs none)	−0.034	−0.052	−0.017	**<0.001**	−0.003	−0.021	0.016	*0.788*
One day lag Snow lying on ground?	−0.017	−0.035	0.000	0.052	−0.011	−0.029	0.008	*0.259*

^a^Univariate associations all adjusted for individual level variables: wear time, age, sex, weight status and weekend/weekday.

﻿For all *p* values, bold font indicates *p* < 0.01, regular font indicates *p* < 0.05, and *italic *font indicates *p* > 0.05.﻿

**Fig. 3:**
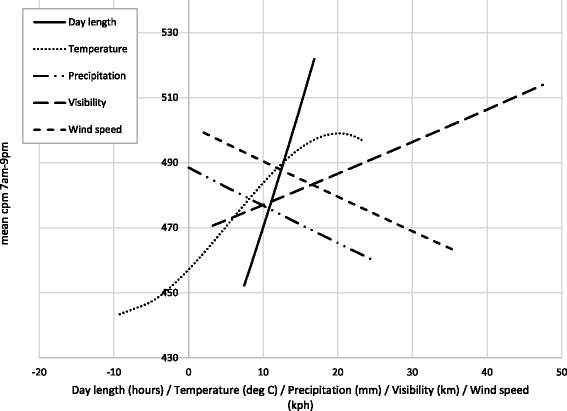
Adjusted mean cpm 7 am-9 pm at centiles (1st-99th) of day length and weather variables. Adjusted for wear time, age, sex, weight status, weekend/weekday, and all other weather/day length variables

The fully adjusted model shows largely the same relationships between cpm and all the weather/season variables as the univariate models. However, after adjustment for temperature and precipitation, the coefficient for the presence of snow is no longer statistically significant. The inclusion of the autocorrelated residual structure in the multilevel model improved the model fit based on a likelihood-ratio test (*p* < 0.05). Residual correlation for measurements taken 1 day apart for the same person on the same measurement occasion were estimated to be 0.115 (95% CI 0.107–0.123). To aid the interpretation of these model outputs, Fig. 3 plots adjusted mean cpm at centiles of each of the weather variables.

**Fig. 4:**
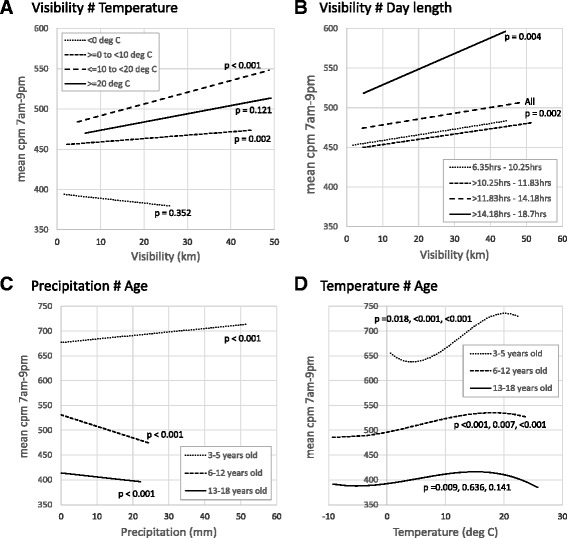
Graphic illustration of interactions between weather variables and demographic factors. All figures show adjusted mean cpm 7 am-9 pm at centiles (1st-99th) of (**a**) Visibility stratified by temperature, (**b**) Visibility stratified by day length, (**c**) Precipitation stratified by age, and (**d**) Temperature stratified by age. All models adjusted for wear time, age, sex, weight status, weekend/weekday, weather variables and day length. *p* values are for regression coefficients in stratified models

Of the 15 pairs of interactions examined (each pairwise combination of the four weather variables, day length, and age), four were statistically significant; Visibility/Temperature, Visibility/Day length, Precipitation/Age and Temperature/Age. Figure 4 shows predicted cpm at mean values of all covariates from stratified models for each of these interaction pairs. Visibility showed a positive relationship with cpm across all bands of temperature (Fig. 4a) and day length (Fig. 4b), except for temperatures below 0 °C where the relationship was not statistically significant. The association appears to be larger (steeper slope) on warmer and longer days. The relationship between precipitation and cpm differed by age group (Fig. 4c). It was positive among 3–5 year olds whereby more precipitation was associated with higher cpm, whilst the relationship was negative for both the older age groups. The effect was greater, with a steeper decline, among 6–12 year olds than 13–18 year olds. The effect of temperature appears to be greatest amongst the youngest children (Fig. 4d) with progressively smaller effects in the older age groups.

### QU2) how do the relationships between day length, weather conditions and physical activity vary between countries?

**Fig. 5:**
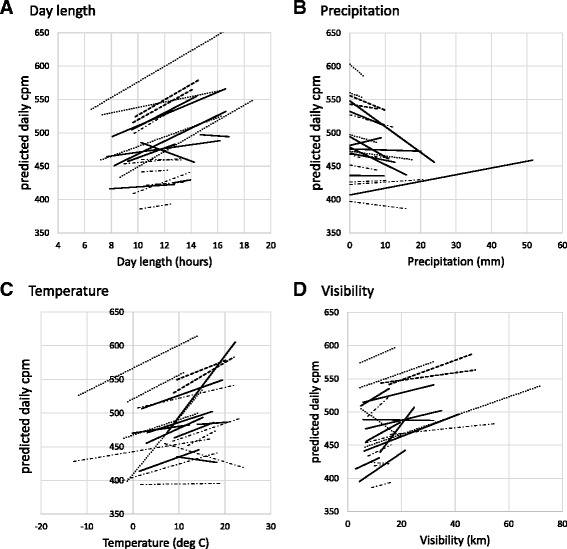
Random slopes and intercepts for (**a**) Day length, (**b**) Precipitation, (**c**) Temperature, (**d**) Visibility, by Setting. Lines are shaded by region ( Northern Europe,  Australia,  Western Europe, and  USA). All lines are plotted between 5th and 95th centile values of the independent variables by setting

Figure 5 shows the random slopes and intercepts for day length, precipitation, temperature and visibility by setting. There are some settings in which day length (Fig. 5a) shows a stronger association with cpm (i.e. a steeper slope) than others, and there is one setting (EYHS Portugal) where the trend is in the opposite direction; cpm increases as hours of daylight decrease.

For precipitation (Fig. 5b) the lines tend to fan in so that those with the higher intercepts (higher mean cpm when there is no rain) show the steepest declines in cpm with increasing rainfall. This observation is also indicated by the negative covariance between slope and intercept terms (−0.0002, *p* = 0.03). Settings with lower intercepts show flatter, or even positive associations between cpm and precipitation. The temperature plot (Fig. 5c) fits with the cubic relationship seen in the main model; settings with higher temperature ranges show flatter or negative associations.

For visibility (Fig. 5d) and wind speed (not shown), the plots show broadly similar associations across the settings in terms of direction of association, with some variability in the slope. The exception to this is one Northern European study (EYHS Denmark, Copenhagen) where the visibility association is steeply negative. In terms of regional trends across these figures, the two Australian studies show broadly similar patterns, and the TAAG cities (plotted individually here) are generally clustered towards the bottom, but there do not otherwise seem to be particular regional differences.

### QU3) in which countries do children appear to most maintain their physical activity levels given the weather conditions they are exposed to?

**Fig. 6:**
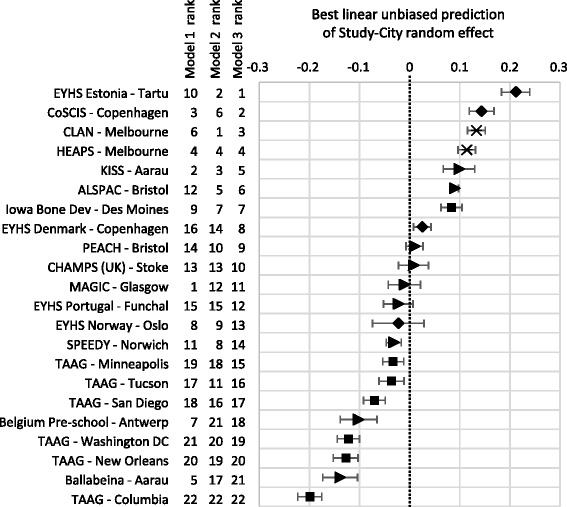
Setting level (random intercept) effects (with 95% CI) on log cpm for fully adjusted 4-level models (as presented in Table 2). Ranks of random effects given for: Model 1 = Null model (no explanatory variables), Model 2 = model with individual level covariates only (wear time, age, sex, weight status, weekday vs weekend day), and Model 3 = Fully Adjusted model (as plotted) with covariates, all weather variables and day length. Markers indicate study location in: ◆ Northern Europe, ► Western Europe, ■ USA, × Australia

Figure 6 shows the setting level random effects on log cpm for the model shown in Table 2. This figure indicates the difference in intercepts between the different study cities, so that values greater than zero indicate higher average log cpm after adjustment, and values less than zero indicate lower average log cpm. Figure 6 includes the rank of studies by random effect for three iterations of the model; the null model with no explanatory variables, a partially adjusted model with the individual characteristics, and the full model with all explanatory variables as presented in Table 2. Looking at the rankings for Model 1, all the TAAG sites are clustered at the bottom (i.e. lower log cpm than the average setting) and the studies in young children (Magic, Belgium pre-school, Ballabeina) are near the top (higher log cpm than the average setting). Adding the individual level covariates (Model 2) moves some of the TAAG sites up closer to the average (especially Tucson and San Diego) while Belgium pre-school, Ballabeina and Magic drop. In the fully adjusted model (Model 3, and plotted values), it is the two Australian studies (HEAPS and CLAN; both located in Melbourne), EYHS Estonia and CoSCIS Copenhagen that do best. Children in these studies have the highest mean log cpm given the day length and weather conditions they experience, considerably higher than the average setting.

## Discussion

This work was prompted in part by the observation that the seasonal patterns in children’s physical activity clearly visible in the UK are not necessarily replicated elsewhere [24]. Our analyses have shown that the relationships between weather conditions and physical activity vary between settings. It appears that children from Melbourne, Australia and Northern Europe that have higher activity levels given the weather conditions they experience compared to those in Western Europe and USA.

The use of a pooled dataset from around the world allowed us to get a clear picture of the relationship between physical activity and different weather variables. Temperature, precipitation, wind speed and visibility are all significantly associated with children’s physical activity across a wide range of exposures. While most weather conditions show broadly linear relationships with physical activity, temperature exhibits a more complex relationship, being associated with increased activity in the range 0–20 °C and decreased activity at higher temperatures. Past studies undertaken in temperate locations have typically reported a straightforward linear relationship [18, 21, 22] of increased temperature associated with increased physical activity. Our results suggest that where mean temperatures sit within the range of 0–20 °C that this is a reasonable assumption. However, we found that mean daily temperatures below 0 °C showed a flatter relationship with physical activity, and those higher than 20 °C were associated with a decline in physical activity. The latter association is similar to that seen in southeast Australia [34] and is likely to be a contributory factor to the reduced activity levels levels seen during high summer in another recent Australian study [35].

In addition to investigating the main effects of different weather conditions, we set out to establish if and how these factors interact with each other. Past research has associated overall climatic conditions with varying physical activity levels in adults [23]. However, we found relatively few significant interactions between the weather conditions, suggesting that combinations of conditions may generally be additive rather than interactive. Of the six interactions between the weather variables, only one was statistically significant (temperature and visibility), and there was one statistically significant interaction between weather and day length (visibility).

Age showed an interaction with two of the four weather variables. This included a significant interaction between age and precipitation. Among pre-school aged children the relationship appeared to be positive, with high rainfall associated with increased physical activity. Few studies have examined the relationships between weather conditions and physical activity among pre-school aged children. Where these associations have been studied, results are inconclusive [36], although there is some evidence that rainfall is associated with decreased physical activity [37]. This age group represents a small proportion of the ICAD sample, and is drawn from a small number of ICAD studies (eight of the 22 settings), so care must be taken in interpreting these findings, but it is suggestive that the impact of weather conditions in younger children’s physical activity may be different to that in older children and adolescents, and that these associations should be investigated further. It may also be instructive for future work to consider other non-climatic effect modifiers. Previous work in the UK has found an interaction between accelerometer-measured physical activity and week/weekend day whereby seasonal patterns are stronger on weekends [12]

Given the large size of our data set, it is perhaps unsurprising that most of the variables we included in our models show a statistically significant relationship with physical activity. But, importantly, the magnitude of the effect of these variables was comparable to that of established correlates of physical activity behaviour in these analyses. Using the fully adjusted model as given in Table 2 to predict cpm across the full ranges of the independent variables shows that a 2 cm increase in rainfall is associated with a slightly larger decrease in cpm (−26.4 cpm) than that associated with being overweight or obese relative to healthy weight (−24.4 cpm), or a year’s increase in age (−25.9 cpm).

Looking at the setting level random effects shows that some settings have higher average cpm after adjustment for individual level covariates and weather variables. In terms of regional trends, studies set in Northern Europe and Melbourne, Australia appear to better than average given the weather conditions they experience. Children in these regions are apparently more active on average (see Figs. 2 and 5), and when exposed to adverse weather conditions (Figs. 5 and 6). The six TAAG sites are all clustered towards the bottom of Fig. 6, indicating lower than average cpm given the characteristics of the participants and the weather conditions experienced. This could indicate a country level effect, the sites being spread across the United States, with potential cultural response to the weather. However, the Iowa Bone Development study, based in Des Moines, sits much further up the Figure.

It is perhaps particularly instructive to compare estimates for settings that appear to have similar weather conditions. Bristol, Norwich, Des Moines and Antwerp all had similar mean temperatures and rainfall, but are ranked quite differently in Fig. 6, with Bristol and Des Moines having higher than average (Bristol-ALSPAC and Des Moines), and average (Bristol-PEACH) cpm given the weather conditions they experience, and Norwich and Antwerp being below average. It could be that children in Bristol and Des Moines are better adapted in some way to these conditions, although differences in the effect between the two Bristol studies (ALSPAC and PEACH) suggest that these differences could be due to the differing samples rather than settings.

Our results have methodological implications for the collection of physical activity data and the comparison of such data between studies and places. Weather conditions are associated with children’s physical activity, and the associations differ between settings. Adjustment should therefore be made for both season and weather when comparing physical activity across settings, and over time. Although it has been suggested that weather and season need not be a concern in all settings in adult populations [38], our findings show that for children, especially pre-school and primary school-aged children, they should both be considered in all settings. The nature of their relationships with children’s physical activity may vary with setting and exposure range, but their impact should be locally investigated before they are ruled out as determinants of behaviour.

This study has a number of strengths and limitations. We were able to use a large, international dataset for which physical activity was measured objectively. The pooling of data within ICAD provided consistent accelerometer processing protocols and weather data were extracted from a similarly standardised data source. The international scope of both datasets allowed the examination of weather and physical activity relationships across a range of locations and therefore a large range of exposures.

In terms of limitations, weather data for each day in each setting were derived from a single weather station and the link between physical activity and weather had to be made on the basis of overall study location rather than participant home location. Although this is a method used in many studies investigating relationships between weather and behaviour [18–22, 25, 39], it limits the precision of weather exposure estimates, and may lead to some attenuation of associations with physical activity. The ICAD data were recorded in a wide range of weather conditions. However the different study samples, and the different times of year over which they were collected, meant that these exposures were not equally distributed across different ages and sexes, and that in some locations measurements were taken over smaller portions of the year than others. For example, some of the warmer study locations were centres in the TAAG study, which investigated physical activity in adolescent girls only. Although our multilevel modelling approach adjusts for these differences to some extent, some of the setting level differences we observe may reflect differences between samples rather than locations. Some studies were based in schools, and we were not able to account for potential school and class level clustering, for example.

Although accelerometers provide an objective measure of physical activity they are not without their limitations. They have a limited ability to assess activity while the wearer is cycling [40], and the units used in ICAD must be removed altogether during aquatic activities. This may have impacted the associations we observed if children’s propensity to undertake these types of activity is associated with weather conditions. Finally, the studies included in these analyses all come from high-income countries, meaning our findings may not be applicable in other countries.

## Conclusions

We find variation in the relationship between weather conditions and physical activity between ICAD studies and settings. There is evidence that children in Northern Europe and Melbourne, Australia are not only more active on average, but also more active given the weather conditions they experience. Further investigations into the nature of physical activity in these regions may therefore be useful to develop strategies to promote sustainable physical activity increases elsewhere. Weather conditions undoubtedly appear to present a barrier to physical activity, particularly among young children. Future work should therefore consider strategies to mitigate such impacts, and interventions involving changes to the physical environment should consider how they will operate in different weather conditions.

## Abbreviations

°C: degrees Celsius
cpm: counts per minute
GSOD: Global summary of the day
ICAD: International children’s accelerometry database
km: kilometre
kph: kilometres per hour
mm: millimetre
MVPA: Moderate to vigorous physical activity
